# Neural-fuzzy machine learning approach for the fatigue-creep reliability modeling of SAC305 solder joints

**DOI:** 10.1038/s41598-023-32460-4

**Published:** 2023-05-26

**Authors:** Dania Bani Hani, Raed Al Athamneh, Mohammed Abueed, Sa’d Hamasha

**Affiliations:** 1 Hijjawi Faculty for Engineering Technology, Yarmouk University, Irbid, 21163 Jordan; 2 Department of Industrial Engineering, Faculty of Engineering, The Hashemite University, Zarqa, 13133 P.O.BOX 330127, Jordan; 3 Intel Corporation, 2775 NE John Olsen Ave, Apt H116, Hillsboro, 97124 USA; 4 Department of Industrial and System Engineering, Samuel Ginn College of Engineering, Auburn University, Auburn, AL 36849 USA

**Keywords:** Engineering, Electrical and electronic engineering, Mechanical engineering

## Abstract

The accuracy of reliability models is one of the most problematic issues that must be considered for the life of electronic assemblies, particularly those used for critical applications. The reliability of electronics is limited by the fatigue life of interconnected solder materials, which is influenced by many factors. This paper provides a method to build a robust machine-learning reliability model to predict the life of solder joints in common applications. The impacts of combined fatigue and creep stresses on solder joints are also investigated in this paper. The common alloy used in solder joint fabrication is SAC305 (Sn–Ag–Cu). The test vehicle includes individual solder joints of SAC305 alloy assembled on a printed circuit board. The effects of testing temperature, stress amplitude, and creep dwell time on the life of solder joints were considered. A two-parameter Weibull distribution was utilized to analyze the fatigue life. Inelastic work and plastic strain were extracted from the stress–strain curves. Then, Artificial Neural Networks (ANNs) were used to build a machine learning model to predict characteristic life obtained from the Weibull analysis. The inelastic work and plastic stains were also considered in the ANN model. Fuzzy logic was used to combine the process parameters and fatigue properties and to construct the final life prediction model. Then a relationship equation between the comprehensive output measure obtained from the fuzzy system and the life was determined using a nonlinear optimizer. The results indicated that increasing the stress level, testing temperature, and creep dwell time decreases reliability. The case of long creep dwell time at elevated temperatures is worst in terms of impact on reliability. Finally, a single robust reliability model was computed as a function of the fatigue properties and process parameters. A significant enhancement of the prediction model was achieved compared to the stress–life equations.

## Introduction

Various types of stresses, such as vibration, thermal shock, mechanical shock, thermal cycling, and tensile and shear stress, are normally considered when the reliability of electronic assembles is explored^1–5^. Mechanical and thermal stresses are commonly associated with electronic components operating under harsh environmental conditions. The thermal cycling process significantly generates shear fatigue and creep loads on the interconnection materials because of the mismatch between the thermal expansion coefficients for the printed circuit board (PCB) and the electronic package. In addition, operating temperature is one of the most significant factors affecting the fatigue resistance of solder joints. However, the failure mechanism in the thermal cycling process is different^6–10^. Different literature models have been commonly utilized to estimate the fatigue resistance of solder joints under different operating conditions, for example, stress-life equation, Coffin Manson, Morrow energy, and Arrhenius models. Al Athamneh et al. investigated the effect of aging conditions and loading levels on SAC-based solder joints in various studies. General empirical equations were generated to define the fatigue behavior for individual solder joints under actual setting conditions. Different levels of experimental conditions and fatigue properties were examined to assess the reliability of the solder joints^11–13^. Siswanto et al. used the Morrow energy model to construct a prediction model for the fatigue life of barrel-type solder joints at different aging temperatures. A negative relationship between aging temperature and solder life was found in the study, where a modified estimation equation was used to illustrate this relationship^9^. Tsou et al. explored the impacts of the strain rate and creep effect that were initiated due to the thermal cycling process by using a finite element model (FEM) simulation. The modified Coffin Manson model with an optimal mesh size was estimated as a prediction model for SAC-based solder joints^14^. The effect of high ambient temperature on the reliability of the solder joints was analyzed in a photovoltaic module by Jiang et al. The fatigue evolutions under thermomechanical cycling environments were examined using FEM and experimental fatigue tests. A prediction equation was constructed as a function of environmental temperatures by utilizing the Arrhenius model. A significant reduction in fatigue life was observed when the ambient temperature was increased to 75 °C^15^. Borgesen et al. demonstrated the damage behavior of lead-free solder joints is complicated and developed a mechanistic model to examine the effects of the interaction between thermal cycling and vibration under various loads^16^.

Developing prediction models for the solder joints’ reliability based on limited studied factor levels might lead to erroneous results. Testing all possible factors that may influence the reliability of the solder joints is extremely difficult because of the large number of experimental combinations, testing cost, and testing time. Thus, accurate reliability models for interconnection materials are essential for many critical applications. Fuzzy logic and artificial neural networks (ANNs) could be employed to enhance the predictability of reliability distributions by increasing the number of experimental combinations and using a new methodology to model reliability^17–19^.

Several studies have employed different types of artificial intelligence tools to improve the predictability and adaptability of fatigue behavior models for microelectronic interconnection materials. Subbarayan and Mahajan investigated the effect of solder joint volume, height, pad size, and other factors on solder joint reliability. General predication models were estimated for solder joint life and profile using a physico-neural approach. The stochastic finite element method was also used to construct an ANN model, and acceptable accuracy with low computational time was achieved from their approach^20^. In another study, an ANN model was developed by Qasaimeh et al. to address the fatigue crack growth pattern of lead-free solder joints during an isothermal fatigue test^21^. Chen et al. demonstrated the effect of the structure parameters (chip thickness, PCB thickness, and solder joint pitches) on the fatigue resistance of solder joints in wafer-level chips. A hybrid model from ANNs and FEM was implemented to assess the reliability of the solder joints^22^.

In the current study, a new method is developed to build a robust machine-learning reliability model to predict the life of solder joints in combined fatigue and creep stress, which is the most common type of stress in many applications. The impact of combined fatigue and creep stress on individual solder joints is also investigated experimentally. Two-parameter Weibull distribution was utilized to perform the reliability analysis for SAC305 solder joints at different stress amplitudes, testing temperatures, and dwell times. Coffin Manson and Morrow energy methods were implemented to demonstrate the fatigue properties of the solder joints under different conditions. An ANN technique was applied to predict the reliability parameters and fatigue properties for the unexamined factor levels. The fatigue properties and operating conditions were combined to form a single predictor of the reliability model using fuzzy logic. A nonlinear optimizer was used to build a robust prediction equation for the characteristic life. Finally, a comprehensive prediction model for the reliability of lead-free solder joints as a function of process parameters and fatigue properties was constructed using ANNs and fuzzy logic. The raw data for this article was extracted from the experimental work that was performed by Abueed to study the effect of the fatigue creep test on SAC305 solder joints reliability^23^.

## Materials and methods

**Figure 1:**
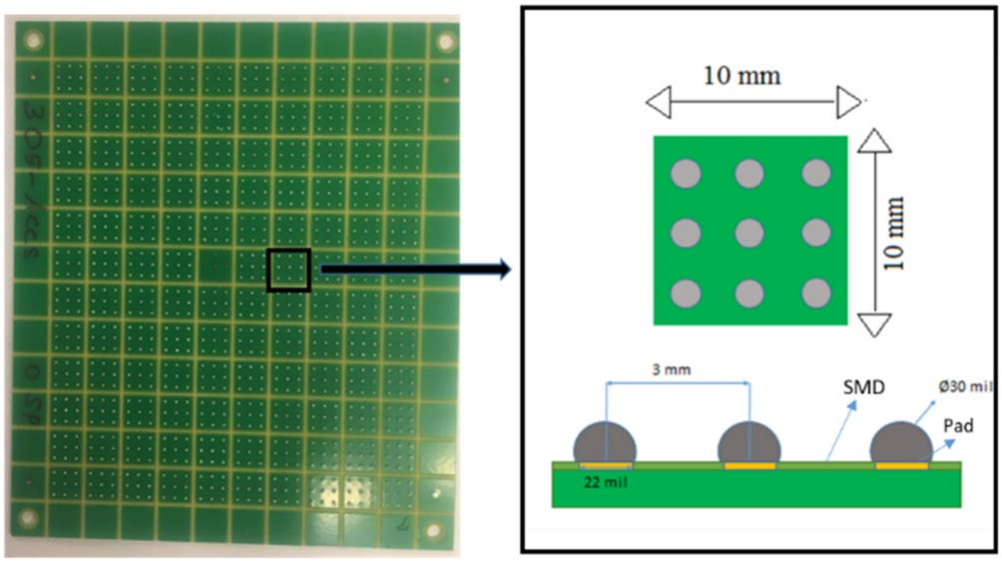
Test vehicle^11^.

**Figure 2:**
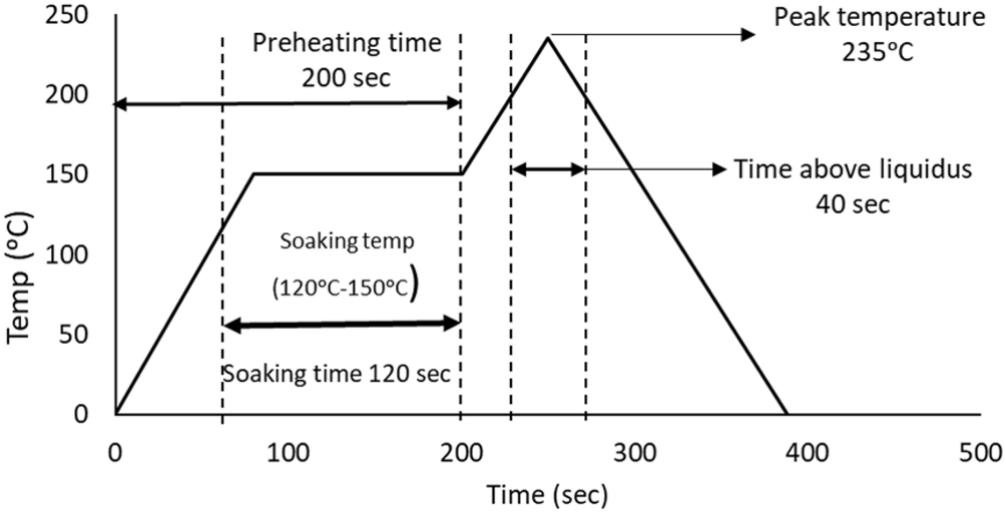
Reflow profile for SAC305 solder joints^11^.

**Figure 3:**
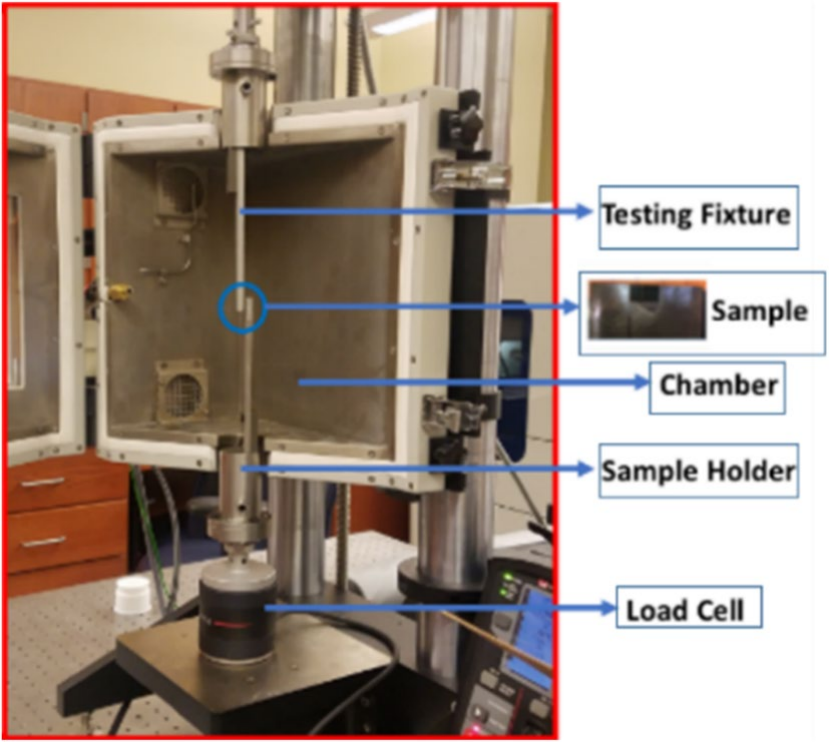
Universal testing machine, Instron 5948^23^.

**Figure 4:**
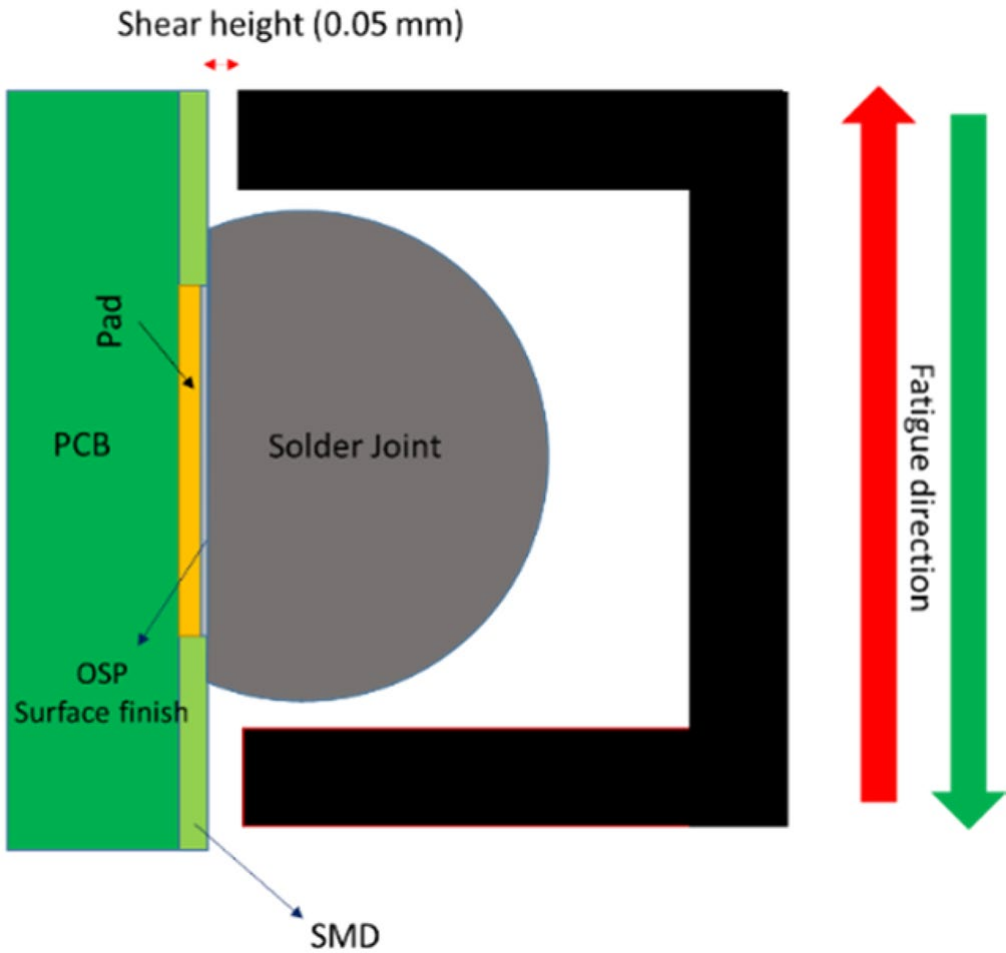
Configuration between the solder joint and the testing fixture^11^.

The fatigue performance and creep resistance of SAC305 solder alloy was investigated using accelerated combined creep and fatigue test on individual solder joints. The test vehicle was an array of SAC305 solder joints (36 × 30) with a pitch distance of 3 mm, a copper pad diameter of 22 mil, and a solder joint diameter of 30 mil. Solder mask defined and OSP (organic solderability preservatives) surface finish were used in the test vehicle fabrication. FR-4 material was used in the PCB manufacturing process. Figure 1 shows the test vehicle design^11^. First, the whole PCB was cut to small parts where each part contains 9 solder joints (3*3). Each array of 3 × 3 solder joints was fixed in the testing machine as one unit, but the test was individually performed for each solder joint. In the first stage of the fabrication process for the test vehicle, a sticky flux was applied on the copper pads using a stencil with a small diameter. Then, the solder balls were installed in the PCB using a stencil with a large diameter. Finally, a ten-zone reflow oven with a nitrogen environment was utilized in the soldering process. The preheating time was 200 s, and the cooling rate was 3.5 °C/s. In addition, the peak temperature reached 235 °C, and the soaking time and time above liquidus were in 120 and 40 s, respectively. Figure 2 represents the reflow profile used for SAC305 solder joints^11^. Figure 3 shows the Instron 5948 universal testing machine, and the experimental setup used to perform the accelerated test^23^. A special experimental setup was designed and manufactured to have an accurate machine setup. Figure 4 illustrates the experimental setup that was used in the test^11^. The fixture was calibrated manually to perform the fatigue creep test on the individual solder joint separately. Two types of stress (fatigue and creep) were applied to the solder joints at the accelerated test under different operating conditions. Three operating factors were investigated for fatigue and creep effects: temperature, stress amplitude, and dwell time. Three levels of stress and testing temperature and four levels of dwell time were considered in the study. Table 1 shows the orthogonal array (L27) implemented to examine the factors that contribute to fatigue life and reliability assessment and modeling^23^. The creep effect is represented by dwell time. Seven replicates were utilized at each factor level. The used shear stress is determined by dividing the applied force on the cross-sectional area that was represented by the area of the copper pad. For example, if a 400-g force is applied on the solder joint, the shear stress equals (16 MPa) which represents the 400-g force divided by the area of the copper pad. The shear strain equals the amount of displacement in the solder joint divided by the radial distance between the center of the solder joint and copper pad.

**Table 1:** L_27_ orthogonal array for fatigue and creep accelerated tests^23^.

Exp (i)	Testing temperature (°C)	Dwell time (s)	Stress amplitude (MPa)
1	25	0	16
2	25	0	20
3	25	0	24
4	25	10	16
.	.	.	.
.	.	.	.
.	.	.	.
.	.	.	.
32	100	60	20
33	100	60	24
34	100	180	16
35	100	180	20
36	100	180	24

**Figure 5:**
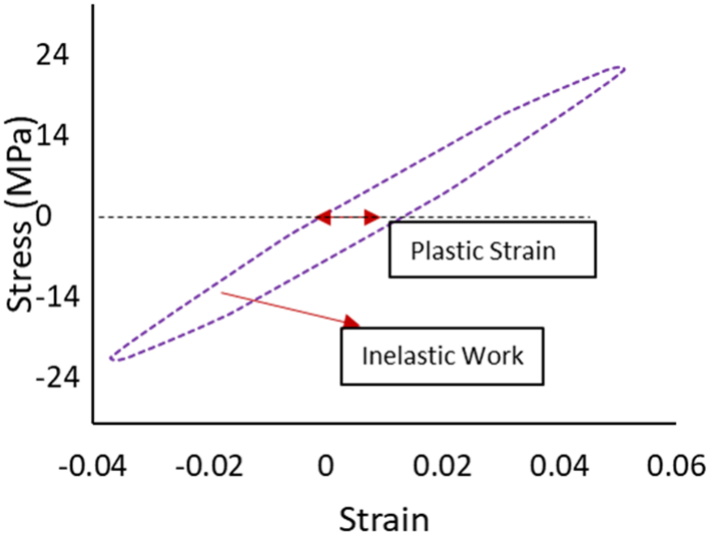
Full hysteresis loop for SAC305 solder joints cycled at 24 MPa (adapted from^11^).

**Figure 6:**
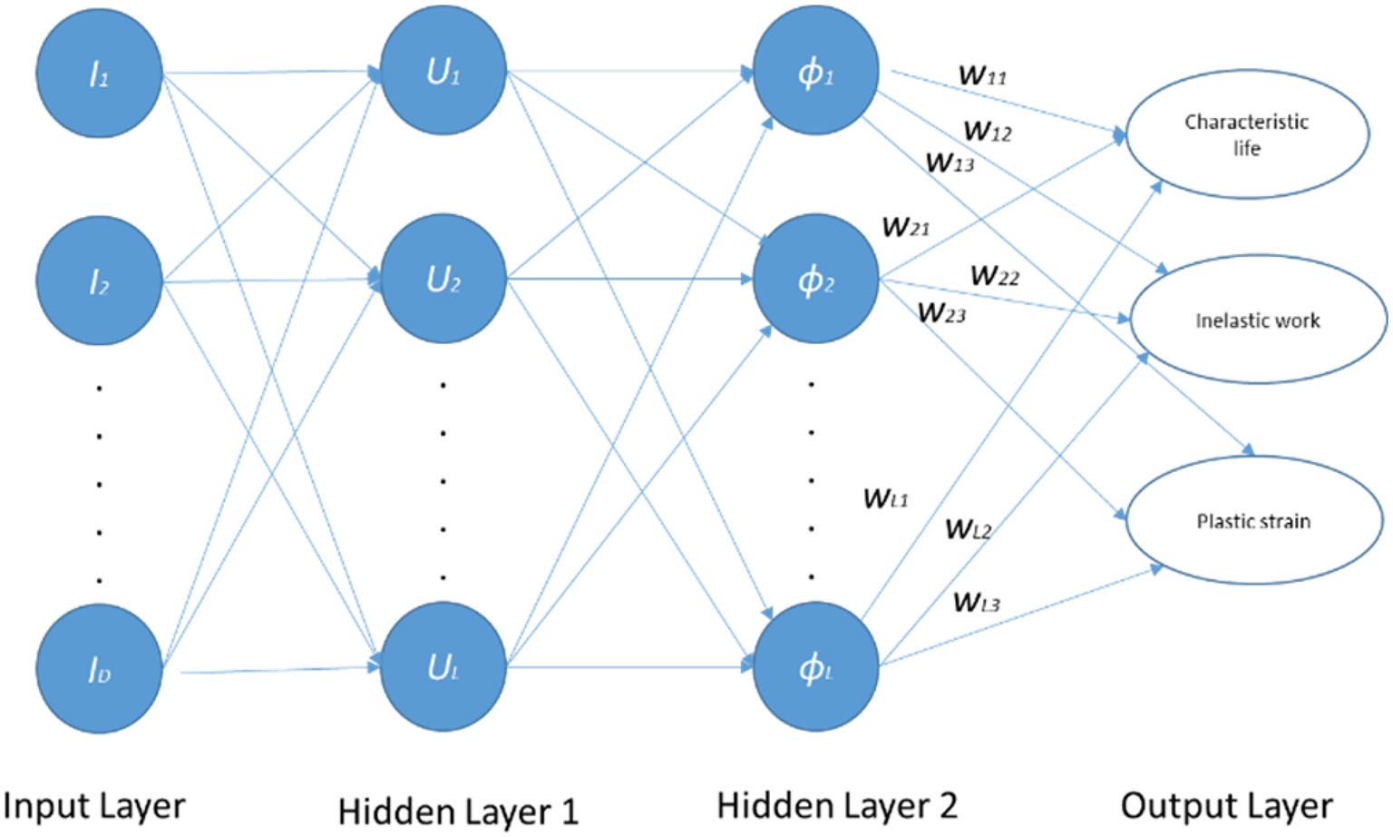
Radial basis function neural network structure^18^.

A two-parameter Weibull distribution was applied for reliability analysis and modeling at each experimental condition. Equation (1) represents the general form of the Weibull reliability density function^24^.

1 $$R\left(t\right)={e}^{-({\frac{t}{\theta })}^{\beta }}$$

where *θ* represents the characteristics life or scale parameter (time when 63.2% of systems will have failed), *β* is the shape parameter (slop of the probability plot), and *t* is the fatigue life in the cycle unit. The stress–life equation (power equation) shown in Eq. (2) is implemented to illustrate the relationship between fatigue life and stress level at different dwell times^25^.

2 $${N}_{63}=U*{J}^{-c}$$

where *J* is the stress amplitude, *N*_*63*_ is the characteristic life, and *U* and *c* are the equation constants. From the stress–strain diagram, the average inelastic work per cycle and plastic strain were determined. In a full hysteresis loop, the area of the stress–strain cycle represents the inelastic work per cycle, and the shift in the stain at zero stress level shows the plastic strain. Figure 5 describes the calculation of plastic strain and inelastic work for SAC305 solder joints cycled at 24 MPa without dwelling and tested at room temperature from the hysteresis loop^11^. ANN was utilized to predict the fatigue properties and characteristic life using the radial basis function neural network (RBFNN) method. Two hidden layers were applied, where the first layer has a Gaussian function, which is called the activator function, and the second layer has a linear function. Five neurons were utilized for each layer. Figure 6 displays the ANN structure (RBF method)^26,27^, where *Is* represents the inputs to the ANN model, and *D* represents the number of inputs. In addition, *U* is the linear function, and *ϕ* is the Gaussian function in hidden layers 1 and 2, respectively. Finally, L and W denote the number of neurons in each hidden layer and the weight, respectively^18^.

**Figure 7:**
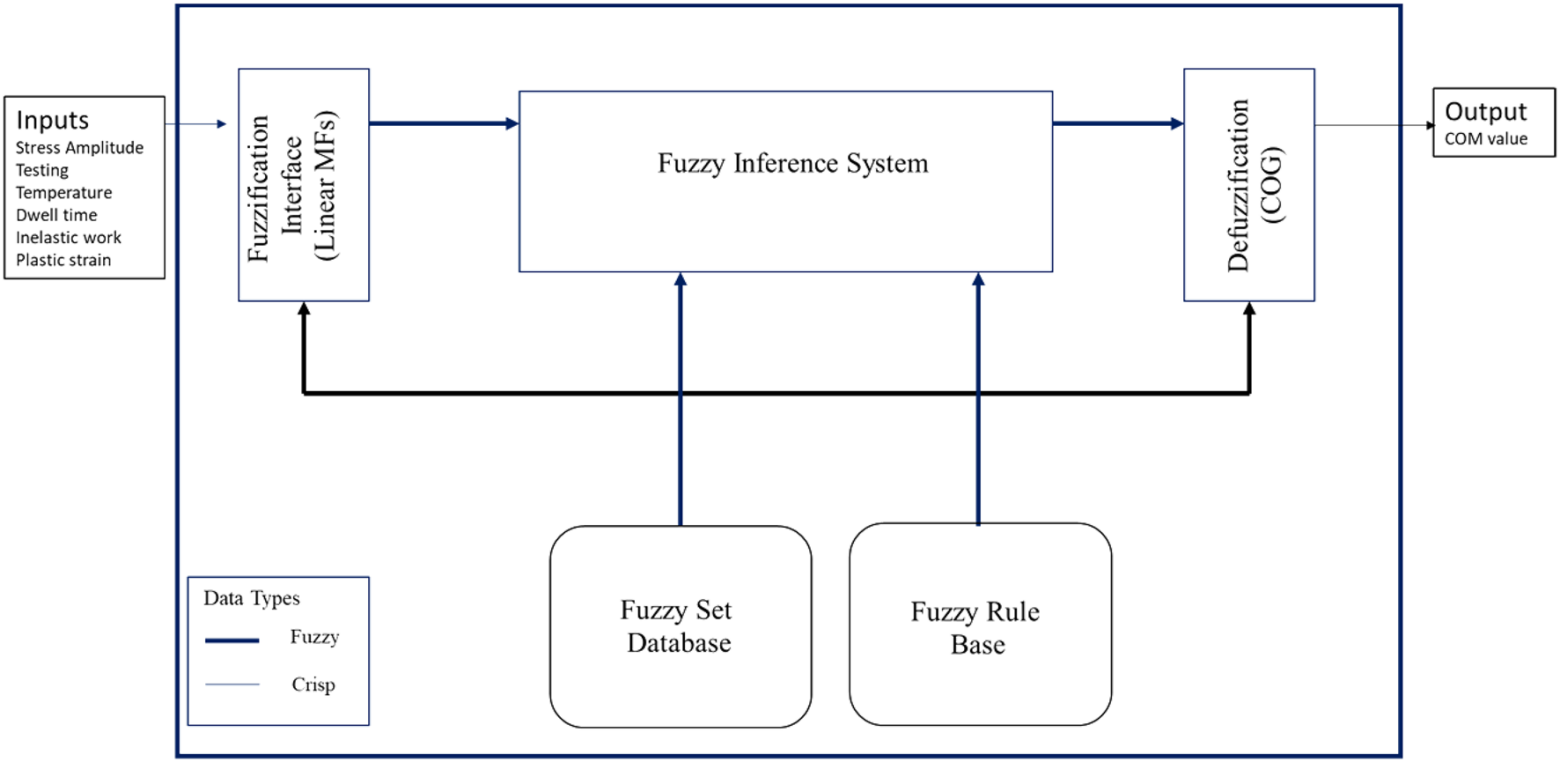
Fuzzy system structure^18^.

**Figure 8:**
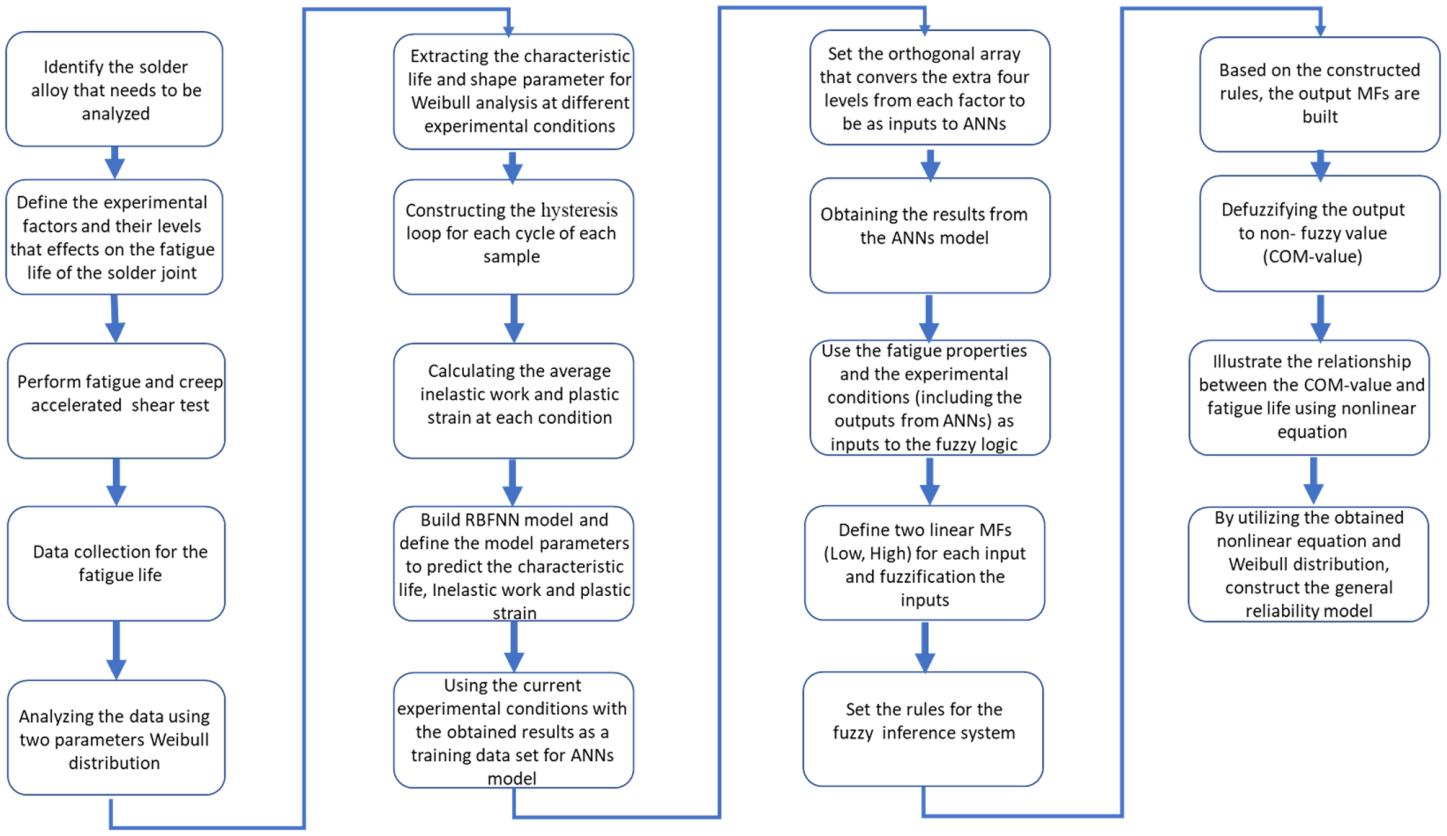
Flowchart of the proposed methodology.

The fatigue properties (inelastic work and plastic strain) and experimental conditions (dwell time, stress amplitude, and testing temperature) were used to build a prediction model for the characteristic life by using fuzzy logic. Theoretically, the fatigue properties can be used to estimate the fatigue life by using different proofed approaches, such as the Coffin Manson and Morrow energy methods. The experimental conditions can be used to predict fatigue life using methods from the literature, such as the stress–life equation and the Arrhenius model. The Mamdani fuzzy method was utilized to convert the multiple predictors for the characteristic life (fatigue properties and experimental conditions) into a single predictor, which is called the comprehensive output measure (COM) value. The five predictors from the operating conditions and fatigue properties were used as inputs to the fuzzy system. Linear member functions (low, high, etc.) were employed as functions for the inputs and outputs of the fuzzy system. Figure 7 shows the structure of the fuzzy inference system^18,19^. The multi predictors were converted into a single predictor using fuzzy systems. The first step involves fuzzification of the inputs using the constructed membership functions (MFs). Rule evaluation is the next step, where the conversion rules of the fuzzy logic are set based on the input’s MF. Then, the output MF is defined in aggregation of the rule outputs using the fuzzy rules. Ultimately, the fuzzy inference output is converted into a non-fuzzy value called the COM value in the defuzzification step. The center of gravity method was utilized as a defuzzifier for conversion in the fuzzy system^28,29^. As an output of the ANNs and fuzzy systems, a single predictor (COM value) was used to construct the prediction model of the characteristic life of the solder joint instead of using five predictors. A non-linear equation is implemented to illustrate the relationship between the COM value and the characteristic life, where the equation constants are determined using Gurobi non-linear optimizer. The obtained equation is substituted in the Weibull reliability equation instead of the characteristic life (scale parameter). The shape parameter value for the general reliability model was estimated by determining the mathematical average of its values under different conditions. Finally, a general robust reliability model was built as a function of the COM value and fatigue life (*t*). Figure 8 shows a flowchart of the general methodology for constructing the general reliability model using the proposed approach. Modeling analysis using the Fuzzy logic and ANNs were achieved using MATLAB R2014b (https://www.mathworks.com/products/matlab.html). All the statistical analysis that were performed in this study were accomplished using MINITAB 16 software (www.minitab.com).

## Results and discussion

### Weibull reliability distribution and ANOVA analysis

**Figure 9:**
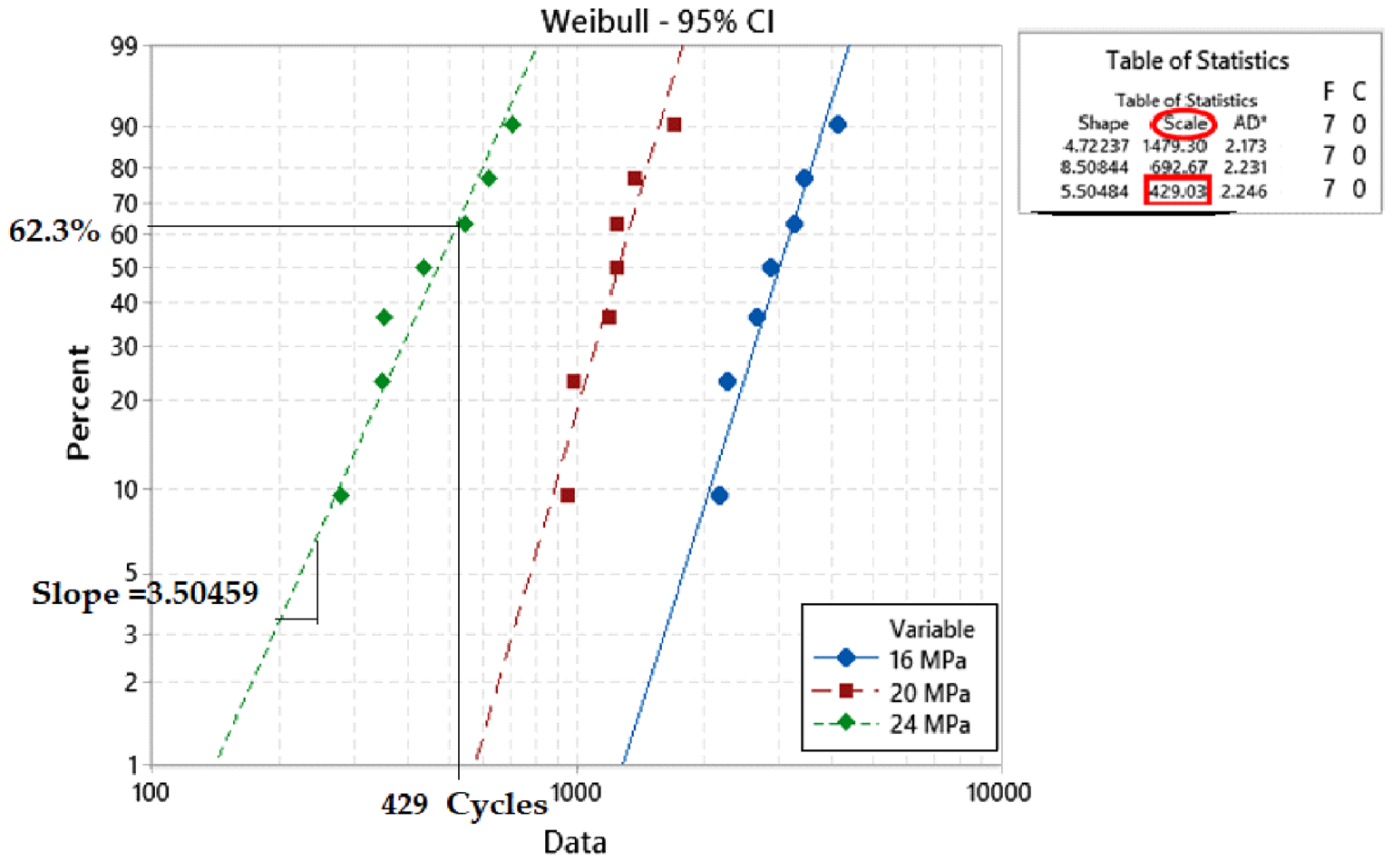
Probability plot of two Weibull distribution for SAC305 solder joints at different stress amplitudes without dwelling, tested at room temperature^23^.

**Figure 10:**
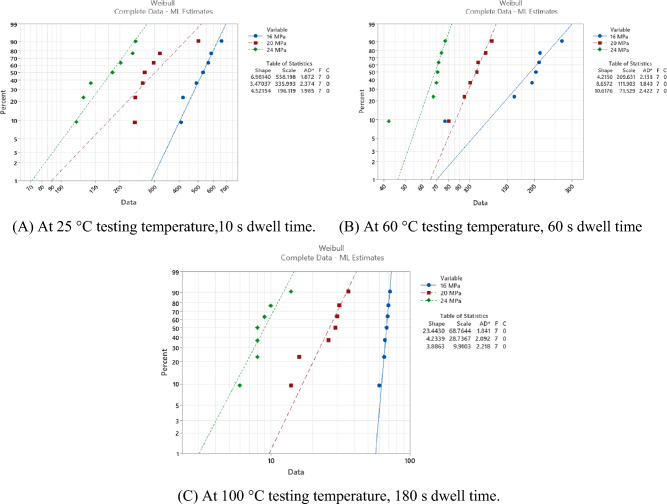
Probability plots for the Weibull distribution of SAC305 solder joints’ fatigue life in different experimental settings.

**Figure 11:**
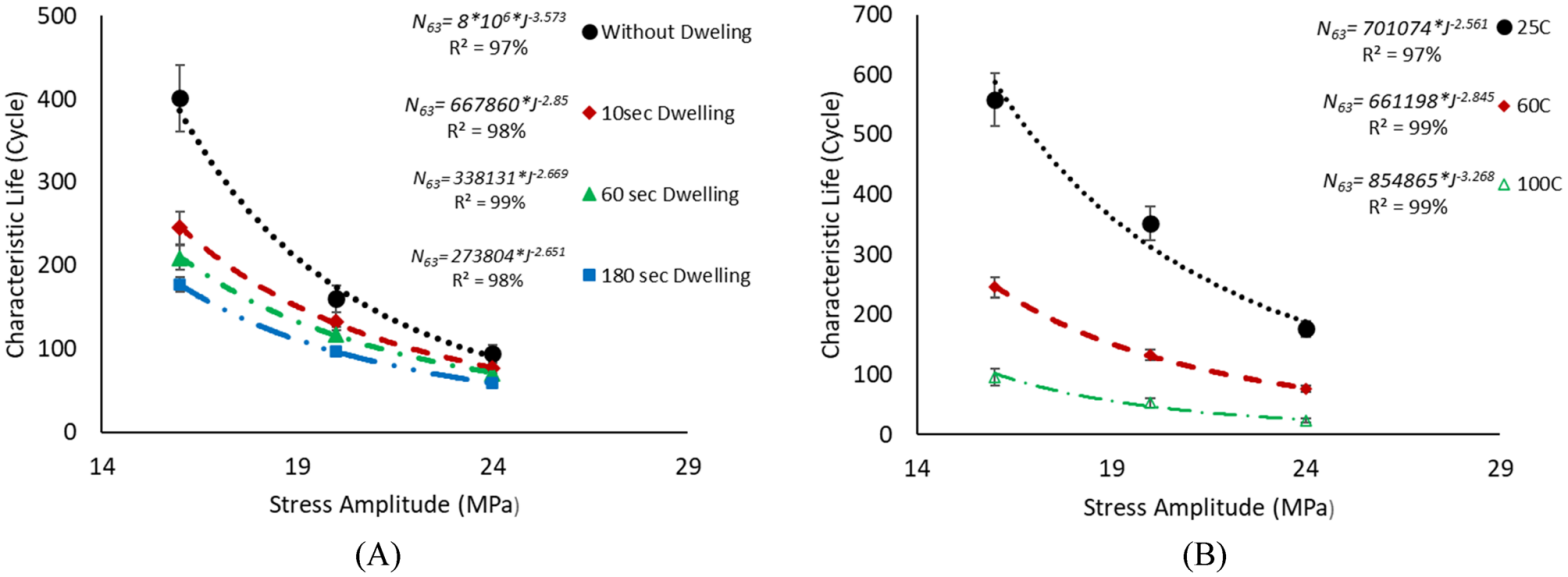
Samples of stress–life relationship for SAC305 solder joints at different conditions: (**A**) cycled at 60 °C testing temperature and (**B**) cycled at a 10 s dwell time.

The experiment was performed with seven replicates in each condition, and the fatigue life data were collected when the solder joints underwent complete failure. A two-parameter Weibull distribution was implemented to analyze the reliability performance at each experimental condition based on the obtained fatigue life. The fatigue life was measured as the number of cycles that the solder joint survived before failure. Failure was defined as the complete fracture in the bulk solder joints or in the intermetallic compound (IMC) layer. The characteristic life and shape parameters were acquired for each condition. The slope of the obtained probability plot represents the shape parameter, and the characteristic life is the number of cycles that 63.2% of the solder joints will have failed. The same failure mode was observed for all tested samples regardless of the applied experimental conditions, where all obtained failures were observed in the solder bulk. Very few failures were observed in the IMC layer, which were excluded from the data analysis and were considered outliers because they were inconsistent with the observed data under the same conditions. Figure 9 shows the probability plot for the Weibull distribution for SAC305 solder joints cycled at different stress levels without dwelling and at 25 °C testing temperature^23^. The probability plots for the Weibull distribution at different stress amplitudes, dwell times, and testing temperatures were plotted, as shown in Fig. 10, to investigate the effect of the experimental conditions on the Weibull distribution parameters^23,30^. MINITAB 16 software was used to perform the statistical analysis. The relationship between fatigue life and stress level at different testing temperatures and dwell times was demonstrated by using the stress–life equation. Figure 11 illustrates the correlation between stress amplitude and characteristic life under different conditions. Figure 11 also shows the stress–life equation and the model adequacy (R-squared). The determined R-squared values for the stress–life model were between 97 and 99%, which represent the robustness of the stress–life equation, even when the creep effect is considered with fatigue. This implies that increasing the fatigue shear load level decreases the fatigue life significantly. By contrast, the contribution of cyclic stress on fatigue life was decreased when the creep effect was present. In addition, the same phenomenon was observed in the relationship between creep stress, testing temperature, and characteristic life, but with different contributions to fatigue life.

**Figure 12:**
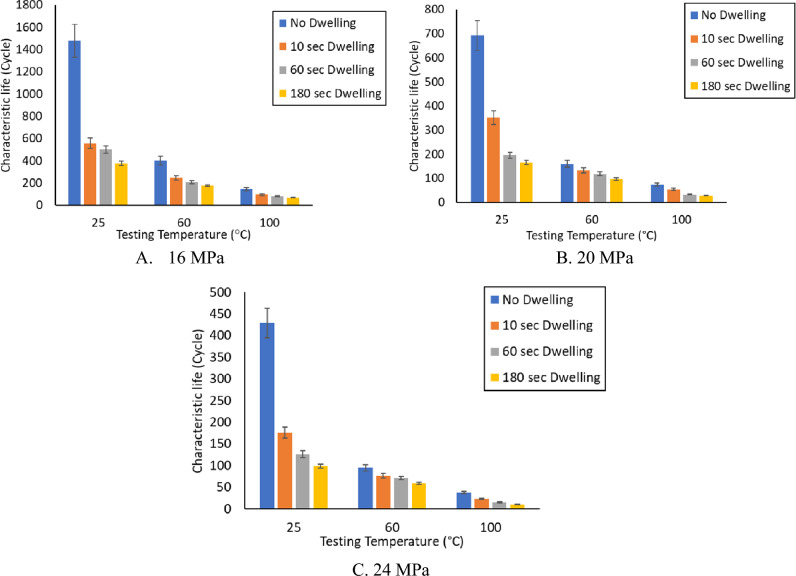
Bar charts showing the effect of the testing temperature and dwell time on the fatigue life of the SAC 305 solder joints at different stress amplitudes.

**Figure 13:**
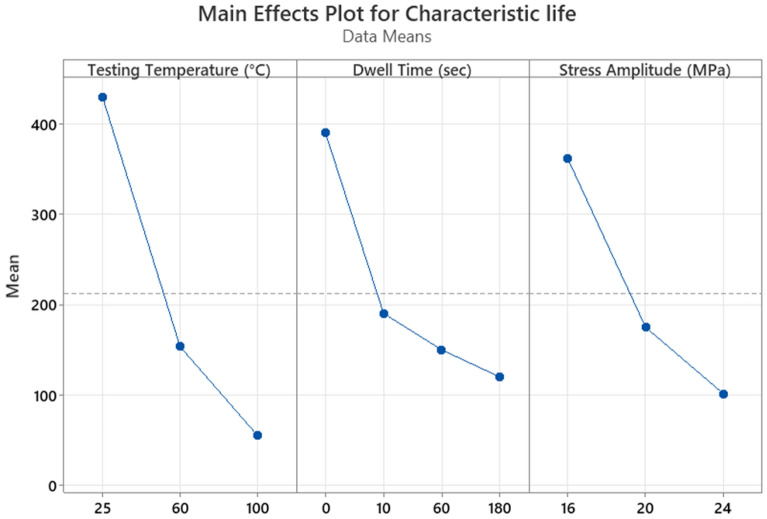
Main effect plot for the characteristic life versus the stress amplitude, testing temperature, and dwell time.

The fatigue data under different conditions are summarized in Table 2. Figure 12 shows a comparison between the effects of the experimental conditions on fatigue life^23^. The smallest fatigue life value (10 cycles) was observed at a 24 MPa stress level, 180 s dwell time, and 100 °C testing temperature. ANOVA was performed to quantify the contribution of each experimental factor. Table 3 represents the ANOVA of fatigue life, and Fig. 13 represents the main effect plots. Regarding, the effect of the testing temperature and dwell time on the fatigue life, increasing the testing temperature had the greatest negative impact on the fatigue life. This effect of the testing temperature was initiated due to precipitate coarsening. The uniform distribution of precipitates on the solder joint body leads to an increase in the overall strength and fatigue resistance of the solder joints. In contrast, the coarsening of the precipitates leads to a reduction in the uniformity of the precipitate distribution, which leads to a decrease in the solder joint strength and fatigue resistance. According to the ANOVA, the effects of all the experimental conditions were statistically significant based on the P-values (< 0.05) but with different contributions. The contributions of stress level, testing temperature, and dwell time on fatigue life were 16.5%, 34.5%, and 15.3%, respectively. By contrast, the creep effect was inflated because the increase in the dwell time had a larger impact on the characteristic life when compared with the effect of the increase in stress level. This conclusion can be determined from the ANOVA and the fatigue data summary as shown in Figs. 12^30^and 13, respectively.

**Table 2:** Weibull distribution parameters for each experimental condition (characteristic life and shape parameter)^23^.

Testing temperature (°C)	Dwell time (s)	Weibull distribution parameters	Stress amplitude (MPa)		
			16	20	24
25	0	Characteristic life	1479	692.5	429
		Shape parameter	4.7	8.5	4.5
60	0	Characteristic life	401	160	95
		Shape parameter	10.2	7	6.1
100	0	Characteristic life	143	73	38
		Shape parameter	7.8	8.1	4.3
25	10	Characteristic life	558	336	196
		Shape parameter	7	3.5	4.5
60	10	Characteristic life	245	133	77
		Shape parameter	13	8	7
100	10	Characteristic life	96	53	23
		Shape parameter	5.9	5.4	7.7
25	60	Characteristic life	501	195.5	126
		Shape parameter	5.1	4.9	13
60	60	Characteristic life	209	112	72
		Shape parameter	4.2	8.6	10.6
100	60	Characteristic life	82	33	15
		Shape parameter	5.4	3	3.4
25	180	Characteristic life	376	165	99
		Shape parameter	5.2	4.3	6.1
60	180	Characteristic life	177	97	59
		Shape parameter	6.2	8	12
100	180	Characteristic life	69	29	10
		Shape parameter	23	4.2	3.9

**Table 3:** ANOVA for determining the effect of the experimental conditions on the characteristic life.

Factors	DF	Seq-SS	Contribution (%)	Adj-MS	F-value	P-value
Testing temperature	2	901,402	34.50	450,701	14.33	0.000
Dwell time	3	400,256	15.32	133,419	4.24	0.014
Stress amplitude	2	430,543	16.48	215,271	6.84	0.004
Error	28	880,648	33.70	31,452		
Total	35	2612,849	100.00

### Inelastic work and plastic strain

**Figure 14:**
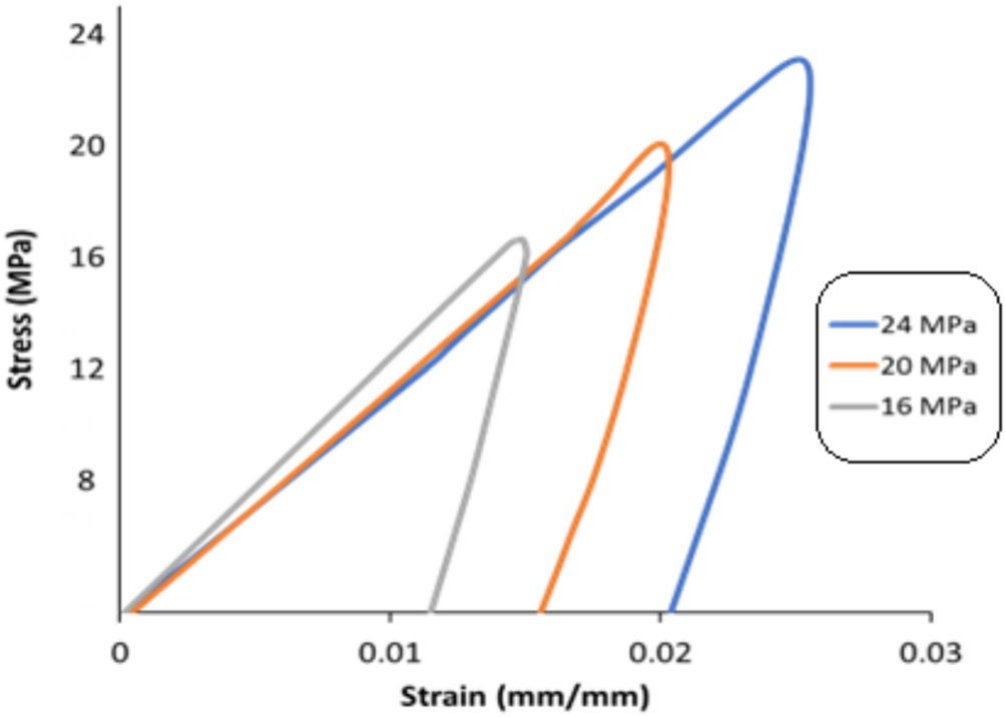
Hysteresis loop of SAC 305 solder joints at different stress amplitudes without dwelling and tested at room temperature^23^.

**Figure 15:**
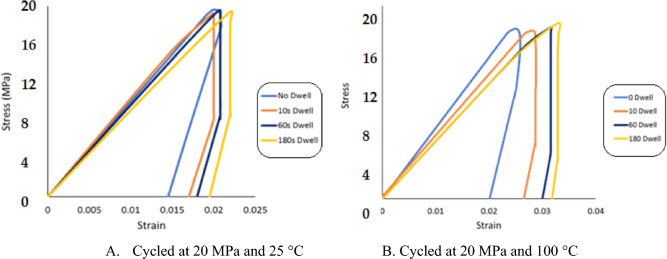
Hysteresis loop of SAC 305 solder joints at different testing temperatures and dwell times and cycled at 20 MPa stress amplitude^23^.

**Figure 16:**
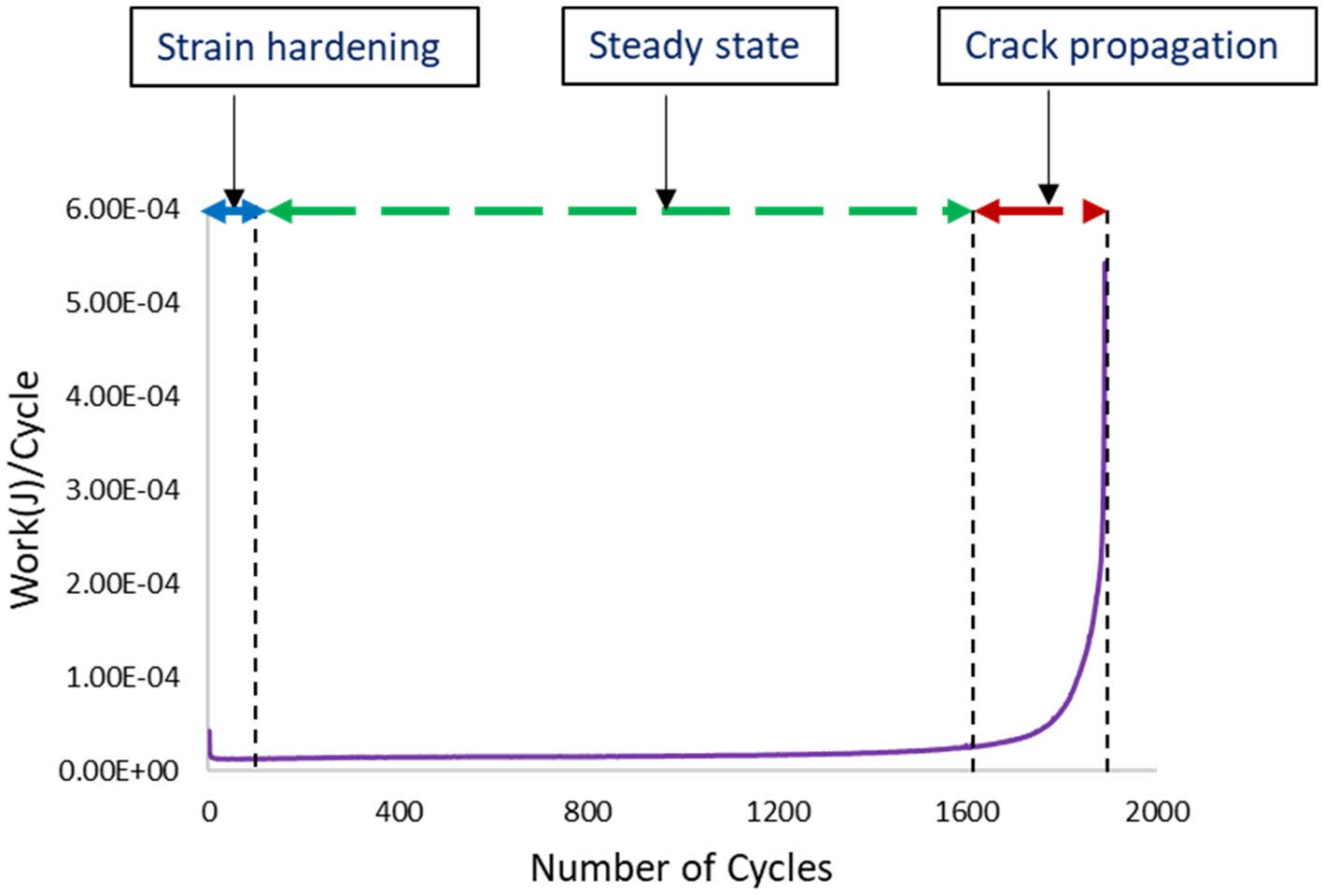
Inelastic work versus the number of cycles for SAC305 solder joints cycled at 16 MPa stress amplitude without dwelling and tested at room temperature (adapted from^12^).

**Figure 17:**
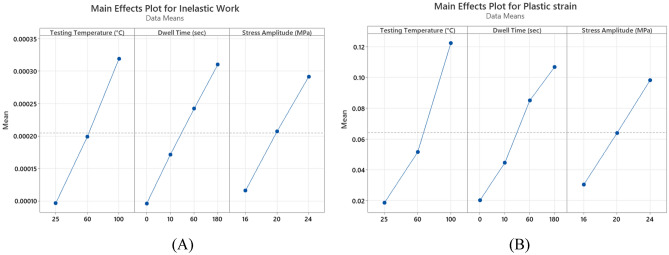
Main effect plots for inelastic work (**A**) and plastic strain (**B**).

The inelastic work per cycle and plastic strain were determined by constructing a stress–strain diagram for each cycle until failure when the cyclic load was applied. The area for the stress–strain cycle represents the inelastic work, and the shift in the strain at zero stress is the plastic strain at that cycle. Figure 14 shows the stress–strain curves for SAC305 solder joints cycled at different stress levels with zero dwell time at 25 °C testing temperature. Figure 15, illustrates the effects of increasing dwell time, stress level, and testing temperature on the hysteresis loop^23^. A notable increase in the stress–strain loop area and a shift in the strain was found when either the stress amplitude and testing temperature or dwell time were increased. The average inelastic work per cycle and average plastic strain were determined for each sample in the steady-state region, as exhibited in Fig. 16^12^. Then, the average values of the fatigue properties were calculated for each condition for the seven replicates. Table 4 shows the values of the average inelastic work per cycle and the average plastic strain under different experimental conditions^23^. To identify the impact of each experimental factor on the fatigue properties, we performed ANOVA, as represented in Table 5 for inelastic work and Table 6 for plastic strain. All the operating factors have a significant contribution on the fatigue properties based on the P-values (< 0.05). The testing temperature had the largest impact on the fatigue properties compared with the load level and dwell time. Figure 17 depicts the main effect plots illustrating the effect of experimental factors on the fatigue properties. After testing temperature, Dwell time was the second largest factor that influenced fatigue properties. Similar results were observed for the ANOVA of the inelastic work and plastic strain in terms of the orders and magnitudes of the factors’ contributions. This is because of the theoretical and mathematical relationships between these fatigue properties.

**Table 4:** Fatigue properties of SAC 305 solder joints under different experimental conditions.

Testing temperature (°C)	Dwell time (s)	Fatigue properties	Stress amplitude (MPa)		
			16	20	24
25	0	Inelastic work (J)	2.45 × 10^–5^	4.45 × 10^–5^	6.69 × 10^–5^
		Plastic strain	0.00499	0.00963	0.01500
60	0	Inelastic work (J)	4.53 × 10^–5^	7.44 × 10^–5^	14 × 10^–5^
		Plastic strain	0.00843	0.01240	0.02600
100	0	Inelastic work (J)	7.94 × 10^–5^	12 × 10^–5^	26.9 × 10^–5^
		Plastic strain	0.01018	0.01898	0.07637
25	10	Inelastic work (J)	4.2 × 10^–5^	6.9 × 10^–5^	11.3 × 10^–5^
		Plastic strain	0.00802	0.01637	0.02041
60	10	Inelastic work (J)	5.68 × 10^–5^	16.3 × 10^–5^	28.1 × 10^–5^
		Plastic strain	0.01398	0.04267	0.05280
100	10	Inelastic work (J)	13.9 × 10^–5^	26.8 × 10^–5^	41.2 × 10^–5^
		Plastic strain	0.04935	0.06923	0.12838
25	60	Inelastic work (J)	6.11 × 10^–5^	11.6 × 10^–5^	14.3 × 10^–5^
		Plastic strain	0.01043	0.02065	0.03181
60	60	Inelastic work (J)	15.2 × 10^–5^	20.7 × 10^–5^	33.9 × 10^–5^
		Plastic strain	0.01642	0.05576	0.12906
100	60	Inelastic work (J)	20.1 × 10^–5^	38.9 × 10^–5^	57.1 × 10^–5^
		Plastic strain	0.07431	0.18665	0.23974
25	180	Inelastic work (J)	9.93 × 10^–5^	20.9 × 10^–5^	17.1 × 10^–5^
		Plastic strain	0.01639	0.02753	0.04192
60	180	Inelastic work (J)	21.7 × 10^–5^	32.1 × 10^–5^	39.4 × 10^–5^
		Plastic strain	0.01798	0.08562	0.15702
100	180	Inelastic work (J)	27.4 × 10^–5^	50.7 × 10^–5^	59.9 × 10^–5^
		Plastic strain	0.13372	0.22063	0.26092

**Table 5:** ANOVA for inelastic work.

Factors	DF	Seq-SS	Contribution (%)	Adj-MS	F-value	P-value
Testing temperature	2	0.000000297	36.30	0.000000149	38.58	0.000
Dwell time	3	0.000000229	27.94	0.000000076	19.80	0.000
Stress amplitude	2	0.000000185	22.60	0.000000093	24.02	0.000
Error	28	0.000000108	13.17	0.000000004		
Total	35	0.000000819	100.00

**Table 6:** ANOVA for plastic strain.

Factors	DF	Seq-SS	Contribution (%)	Adj-MS	F-value	P-value
Testing temperature	2	0.06750	37.97	0.033748	22.81	0.000
Dwell time	3	0.04114	23.14	0.013713	9.27	0.000
Stress amplitude	2	0.02769	15.58	0.013847	9.36	0.001
Error	28	0.04143	23.31	0.001480		
Total	35	0.17776	100.00

### Artificial neural networks and fuzzy logic

The main purpose of implementing the ANN technique is to increase the predictability and accuracy of the proposed model. ANN was utilized to predict the inelastic work, plastic strain, and characteristic life for the unexamined factor levels based on the orthogonal array and the experimental results. After completing the experiments and collecting the fatigue properties and fatigue life data, the test matrix, fatigue life, and fatigue properties, as shown in Tables 1, 2, and 4, respectively, were used as training data for the ANNs. RBFNNs were applied as method coordinators for ANNs with two hidden layers and five neurons for each layer. Extra levels from each experimental condition were considered for the unexamined factor levels. The investigated unexamined factor levels were chosen based on the studied factor levels, where one additional level was taken between each pair of the experimental levels for each factor. This is because the ANN model works more efficiently when the proposed factor levels for prediction are within the training data. The levels that were selected to be predicted were in the middle distance between the pairs of studied levels. In contrast, the ANNs had difficulties and deficiencies when the prediction zone was outside the training data zone. Table 7 shows the orthogonal array for the uncovered factor levels that need to be predicted. The given orthogonal array was used as an input for the ANN model. The results of the ANN prediction model and the experimental results are displayed in Table 8.

**Table 7:** Input orthogonal array to the RBFNNs.

Run (i)	Testing temperature (°C)	Dwell time (s)	Stress amplitude (MPa)
1	40	5	18
2	40	5	22
3	40	35	18
4	40	35	22
5	40	120	18
6	40	120	22
7	80	5	18
8	80	5	22
9	80	35	18
10	80	35	22
11	80	120	18
12	80	120	22

**Table 8:** Results from RBFNN model.

Run (i)	Testing temperature (°C)	Dwell time (c)	Stress amplitude (MPa)	Characteristic life (cycle)	Inelastic work (J)	Plastic strain
1	40	5	18	729.4	2.58 × 10^–5^	0.00636
2	40	5	22	407.3	8.76 × 10^–5^	0.00919
3	40	35	18	300.5	7.01 × 10^–5^	0.00723
4	40	35	22	157.6	16.27 × 10^–5^	0.02569
5	40	120	18	153.8	14.86 × 10^–5^	0.02760
6	40	120	22	124.7	19.83 × 10^–5^	0.04544
7	80	5	18	98.2	14.33 × 10^–5^	0.04328
8	80	5	22	50.5	42.68 × 10^–5^	0.11193
9	80	35	18	100.6	18.45 × 10^–5^	0.06999
10	80	35	22	18.1	45.04 × 10^–5^	0.13620
11	80	120	18	113.6	22.48 × 10^–5^	0.08239
12	80	120	22	22.6	55.25 × 10^–5^	0.23278

**Figure 18:**
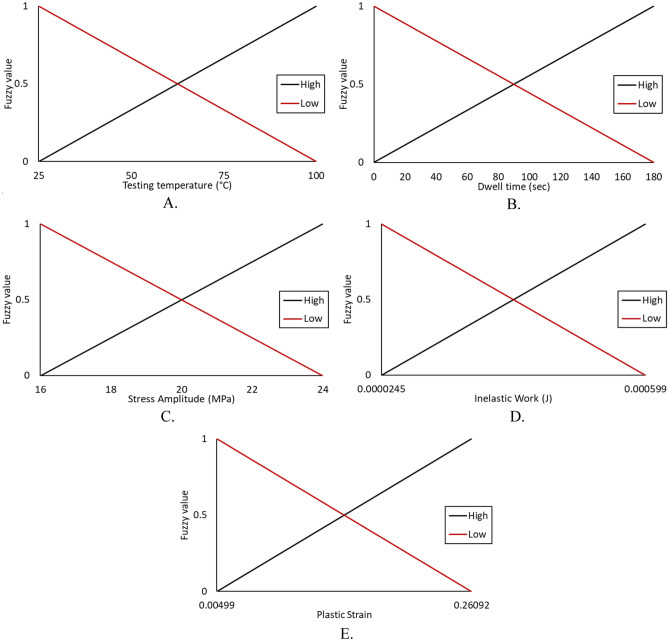
MFs for the inputs of the fuzzy inference system.

**Figure 19:**
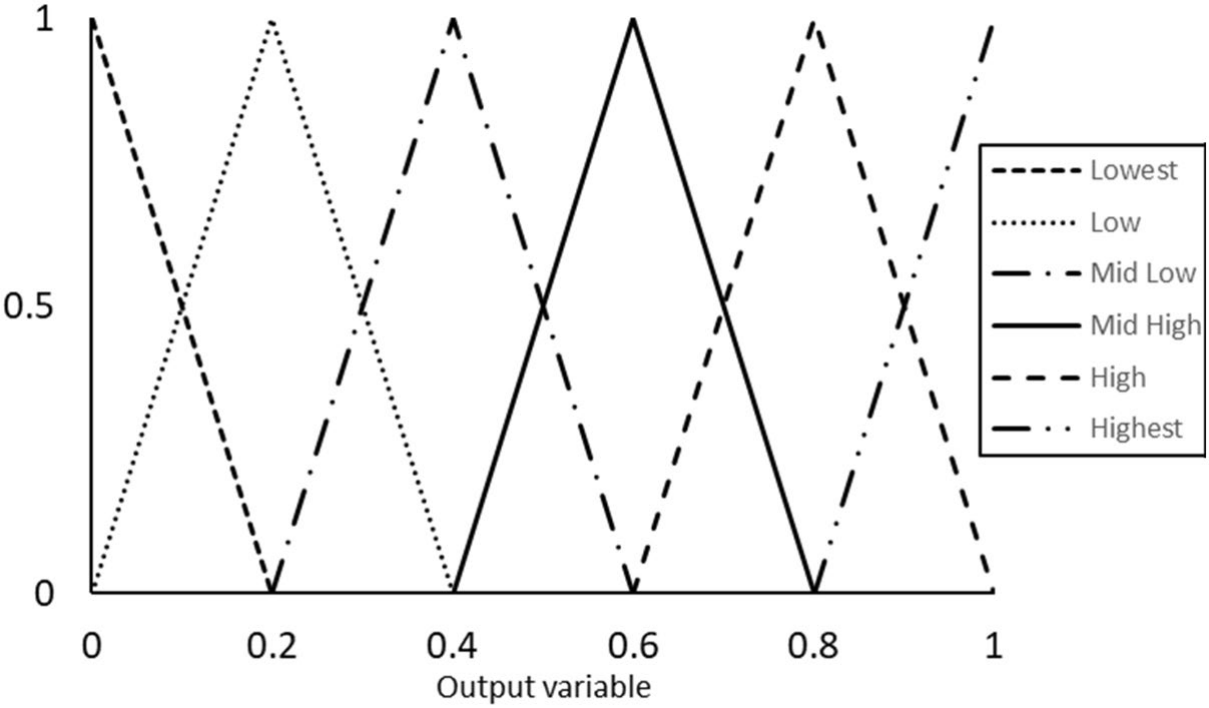
Output MFs for the proposed prediction model for the fatigue-creep life of SAC305 solder joints.

**Figure 20:**
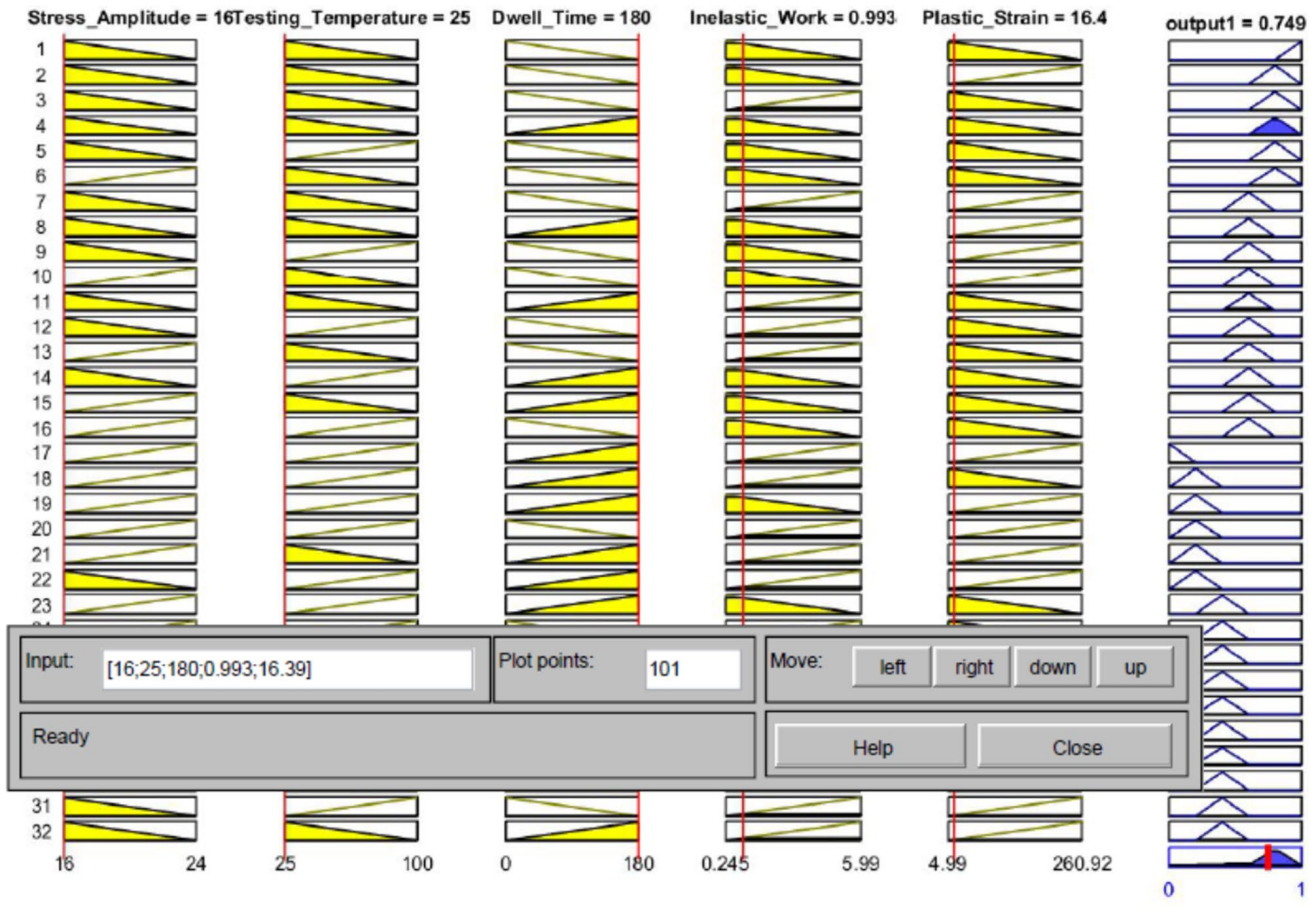
Defuzzification method of the fuzzy model for SAC 305 solder joints cycled at 16 MPa stress level with 180 s dwell time and 25 °C testing temperature.

**Figure 21:**
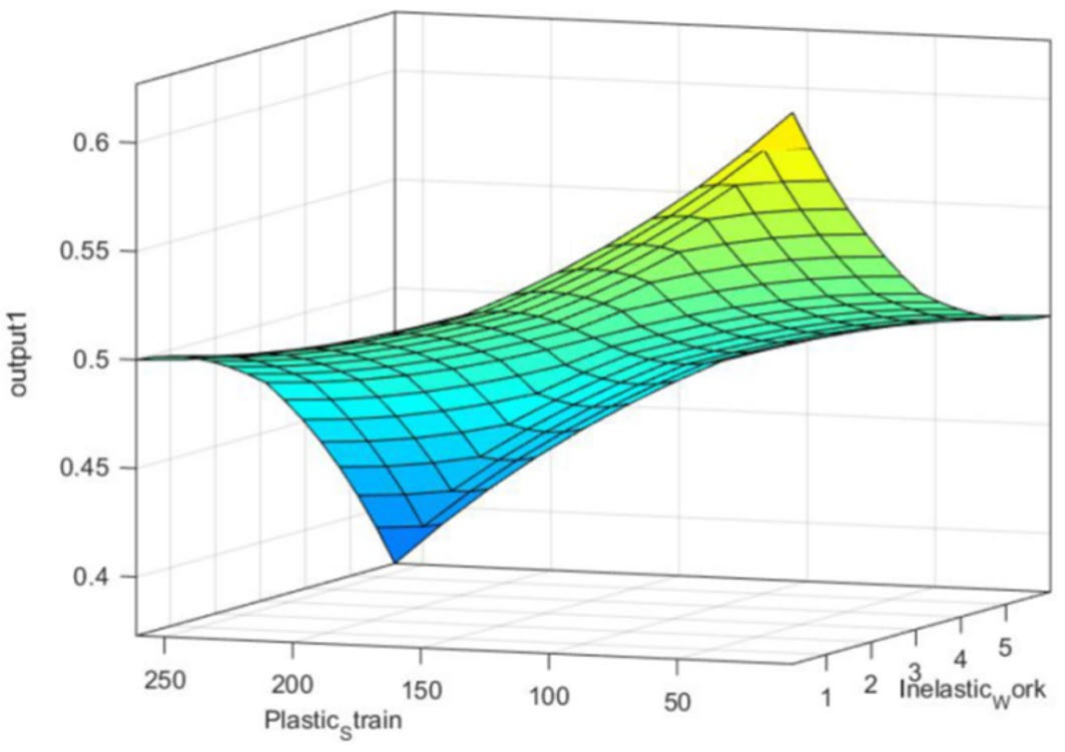
Relationship between inelastic work, plastic strain, and COM value.

According to the different theoretical models, such as the Coffin Manson model, Morrow energy model, stress–life equation, and Arrhenius model, different prediction models can be constructed to predict the fatigue life. These models used the experimental conditions and fatigue properties as predictors and showed robustness in assessing the fatigue life for the solder joints. In contrast, only one independent variable can be utilized for each equation from the mentioned models, which means that five different models with different independent variables can be utilized to predict fatigue life with different accuracies. Using these models in fatigue life prediction results in different predicted fatigue lives under the same conditions, which may lead to the reduced reliability of the results for these models. Conversely, creating a prediction model for the characteristic life as a function of the five different variables has complexity and difficulties in terms of defining the function for each variable and optimizing the equation constants. Therefore, fuzzy logic was utilized to convert the studied variables (fatigue properties and experimental conditions) into a single independent variable, namely, COM value (the output from the fuzzy inference system). The first step in fuzzy logic is input fuzzification. The fatigue properties and operating conditions were defined as inputs to the fuzzy inference system. Two linear MFs were defined for each fuzzy input: low and high. The minimum and maximum values from each input were considered a range for the MFs. Figure 18 shows the MFs for each fuzzy input. The fuzzification step was completed by normalizing the actual value of the inputs to be between 0 and 100% for high MF and between 100% and 0 for low MF. After the fuzzy values for each condition were obtained, 32 fuzzy rules were established based on input MFs in the rule evaluation stage, as shown in Table 9. According to the specified fuzzy rules, six output MFs were defined in the aggregation of the rules’ steps. Figure 19 shows the output MFs. Finally, the center of gravity technique was implemented as a defuzzification method to convert the fuzzy values of the output into a non-fuzzy value (COM value), as displayed. Figure 20 shows the COM value based on specific rules using a fuzzy inference system at 16 MPa stress amplitude, 180 s dwell time, 25 °C testing temperature, 9.93 × 10^–5^ (J) inelastic work, and 0.01639 plastic strain. Table 10 represents the COM values at different fatigue properties and operating condition values. Figure 21 demonstrates the relationship between COM values, inelastic work, and plastic strain.

**Table 9:** Fuzzy rules for the SAC 305 solder joint prediction model.

Rule #	Dwell time	Stress amplitude	Testing temperature	Average inelastic work	Average plastic strain	Output
1	Low	Low	Low	Low	Low	Highest
2	Low	Low	Low	Low	High	High
3	Low	Low	Low	High	Low	High
4	Low	Low	High	Low	Low	High
5	Low	High	Low	Low	Low	High
6	Low	Low	Low	High	High	Mid high
.	.	.	.	.	.	.
.	.	.	.	.	.	.
.	.	.	.	.	.	.
.	.	.	.	.	.	.
27	High	High	High	Low	Low	Mid low
28	High	Low	High	High	High	Low
29	High	High	Low	High	High	Low
30	High	High	High	Low	High	Low
31	High	High	High	High	Low	Low
32	High	High	High	High	High	Lowest

**Table 10:** Determined COM values at different operating conditions.

Stress amplitude (MPa)	Testing temperature (°C)	Dwell time (s)	Average inelastic work (J)	Average plastic strain	COM-value
Experimental conditions					
16	25	0	2.45 × 10^–5^	0.00499	0.937
16	60	0	4.53 × 10^–5^	0.00843	0.81
.	.	.	.	.	.
.	.	.	.	.	.
24	60	180	39.4 × 10^–5^	0.15702	0.35
24	100	180	59.9 × 10^–5^	0.26092	0.063
Predicted conditions					
18	40	5	2.58 × 10^–5^	0.00636	0.772
22	40	5	8.76 × 10^–5^	0.00919	0.707
.	.	.	.	.	.
.	.	.	.	.	.
18	80	120	22.48 × 10^–5^	0.08239	0.522
22	80	120	55.25 × 10^–5^	0.23278	0.364

The relationship between the COM value and the characteristic life was illustrated by using a non-linear equation (Eq. 3), where *k*_*1*_ to *k*_*6*_ are the equation constants, *C* is the COM value, and *N*_*63*_ is the characteristic life.

3 $${N}_{63}={k}_{1}+{K}_{2}*C+{{K}_{3}e}^{C{k}_{4}}+{{K}_{5}C}^{{K}_{6}}$$

**Figure 22:**
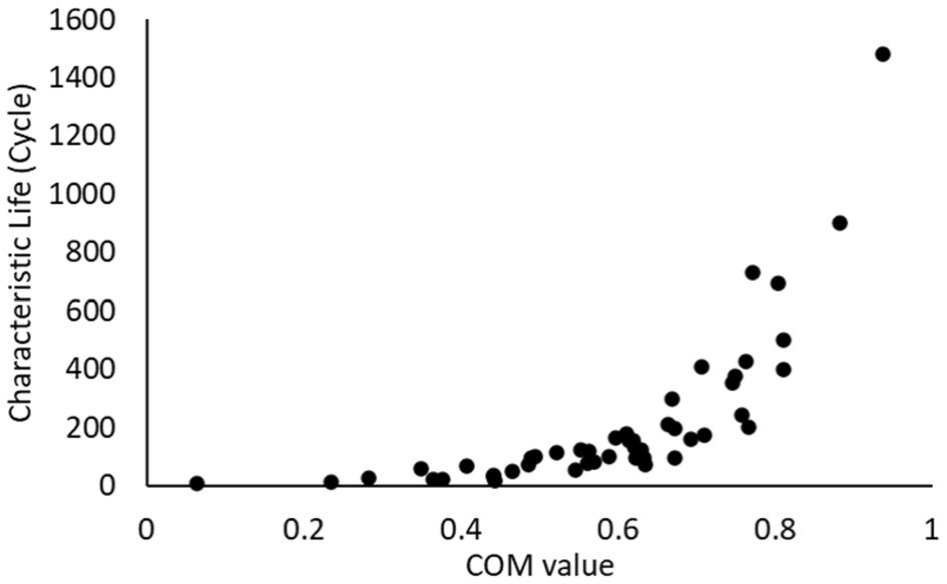
Characteristic life versus the COM value of the final outcomes from the proposed method.

Figure 22 shows the relationship between the COM value and fatigue life. The prediction equation was established based on the observed behavior between the characteristic life and COM value in Fig. 22. The linear term in the equation was used to represent the relationship between the independent variable (COM value) and the dependent variable (*N*_*63*_) when the COM value had a low value. When the independent variable had a medium value, the exponential term was used. A power term was implemented to display the characteristic life behavior when the high values of the independent variable were presented. The final prediction equation was determined in Eq. (4) by using a nonlinear optimizer to estimate the equation constants. The high R-squared value (94%) obtained for Eq. (4) indicates an acceptable goodness of fit index for the suggested prediction equation. To build a robust reliability model, we substituted the prediction equation in the Weibull reliability model (Eq. 1) instead of the scale parameter. The shape parameter was estimated by calculating its mathematical average for all experimental conditions. Equation (5) shows the final general reliability model for SAC305 solder joints as a function of the COM value under different operating conditions and fatigue properties. A logical behavior was not observed for the shape parameter when the operating conditions were changed. The shape parameter was estimated by determining the overall mathematical average of the shape parameter values for the experimental conditions.

4 $${N}_{63}=23.67+12.38*C+{0.61e}^{8.18*C}+{20.74C}^{1.51}$$

5 $$R\left(t\right)={e}^{-({\frac{t}{{N}_{63}=23.67+12.38*C+{0.61e}^{8.18*C}+{20.74C}^{1.51}})}^{6.9}}$$

## Conclusion

The main aim of the study was to develop a robust reliability model to predict the fatigue life of SAC305 solder joints under different experimental conditions. Shear fatigue coupled with creep stresses at different levels of stress amplitude and dwell time was applied using a universal multi-testing machine. The effect of increasing the stress level, dwell time, and testing temperature on fatigue life was investigated by examining three levels from each factor. Negative relationships were found between fatigue life and the studied factors. The characteristic life and shape parameters were determined at each condition using the Weibull reliability analysis. The highest value of fatigue life (1479 cycles) was found for the solder joints cycled at 16 MPa stress level, zero dwelling time, and 25 °C testing temperature. From the stress–strain diagram, the inelastic work and plastic strain were identified under each experimental condition.

ANNs and fuzzy logic were employed to construct the proposed model. The ANN technique was applied to increase the predictivity of the proposed model by increasing the dataset used to build the proposed model. The different studied predictors were converted into a single independent variable (COM value) using fuzzy logic. The relationship between the characteristic life and the outcomes from the fuzzy model was demonstrated using a general prediction equation. An acceptable model adequacy (R-squared) index was obtained from the proposed model. The obtained equation was used to construct a comprehensive reliability model as a function of fatigue life and COM value. However, the obtained model has some limitations. The suggested reliability model has some difficulties and lacks accuracy when the predicted fatigue life was found at conditions that are out of the range of the studied experimental conditions. Furthermore, the reliability model was constructed under the assumption that the behavior of the fatigue-life data follows a Weibull distribution.

## Data Availability

The datasets used and/or analyses during the current study available from the corresponding author on reasonable request.
